# Abstracts from German Journals

**Published:** 1872-10

**Authors:** Ch. Raushenberg

**Affiliations:** Atlanta, Georgia


### ABSTRACTS FROM GERMAN JOURNALS.

BY CH. RAUSHENBERG, M.D., ATLANTA, GEORGIA.

### *THE OCCURRENCE OF MICROSPORON SEPTICUM (KLEBS) IN ACUTE SEPTIC FEBRILE DISEASES.*

Dr. Orth, who is engaged in a series of yet unfinished investigations on this subject, in consideration of its great practical importance, gave, at the meeting of the Berlin Medical Society, March 19th, 1872, the results of his observations in three cases: two were those of new-born infants, whose mothers both suffered with puerperal fever; the third a case of septicæmia, in consequence of an amputation of the thigh.

In the first case, the child died three days after birth of pleuritis in the right side, with an abscess the size of an English pea in the right lung, and one the size of a pin-head in the left. The microscopic investigations showed the presence of very small round bodies, with molecular movements, in the pleuritic exudate. Similar bodies were discovered in the alveoli, the bronchial tubes, and particularly in the blood-vessels of the lungs, in great abundance, while the above-mentioned abscesses did not contain any.

The most interesting part of this case rests in the solution of the question, how the spores of these fungi found their way primarily into the body of this infant. The child died the third day; the umbilicus had not separated itself, and the entire region and the umbilical vessels were perfectly normal, while the mother, at the time of the birth of the child, had considerable fever, and continued to have it. The most natural conclusion is, *that these organisms wandered within the placenta, from the blood of the mother to that of the child,* from which they went with the blood-current into the lungs, and found their way into the pleural cavity. The death of the child must have been caused more by the high grade of fever than by the local morbid process, as the inflammatory phenomena in the lungs and pleura were very moderate.

The second case presented a different character. The child died when seven days old, and while its mother was afflicted with a very severe fever. Extensive inflammation of the umbilical vessels, particularly the umbilical vein, with a perivascular abscess on the inferior surface of the liver, in the sulcus of the venous duct of Aurantius, explained very plainly the route the investing organisms were traveling. The contents of the abscess spoken of consisted of bad pus, representing a grayish-yellow, sanious mass, containing the same tufts of fungi; the contents of the vein itself were of the same character.

Dr. Orth used this opportunity to submit the spores of these fungi, in a moist chamber, as it has been used by microscopists for that purpose, to those conditions which are necessary for their growth and development; he cultivated them to determine their specific character, as Rindfleisch and others have done. The organisms which he raised consisted invariably of single or of pairs of small, roundish, bright-shining bodies, oscillating to and fro, showing very small differences in size. He wants these organisms well separated from the common vibriones accompanying the decomposition of organic substances, which, he says, he never observed in his preparations, when the usual cautions to keep them clean had been scrupulously observed.

A case of septicæmia after amputation of the thigh came next to his observation. All internal organs were pale, anæmic—no abscesses or purulent inflammations of serous membranes perceptible. The surface of the amputation-wound was discolored, of a putrid odor—covered in many places with a whitish-gray coating, which could be torn off in shreds—in others with grayish-green masses, formed by decomposed muscular tissue; the stump œdematous, the muscles interspersed with abscesses, the inguinal glands enlarged and indurated; thrombosis, to any extent, existed neither in the femoral artery nor vein; the veins still contained fluid blood.

The substances coating the wound contained vibriones from decomposition, and also the above-described fungi. Of this substance, sufficient quantities were injected into the abdominal cavities of one rabbit and four guinea-pigs, with Pravaz's syringe. The results did not exactly correspond with previous expectations. Three guinea-pigs remained well; the rabbit, however, and one of the pigs died with peritonitis, respectively in twenty-four and thirty hours. The peritoneum, microscopically examined, contained only the same fungi and their spores.

The microscopic examination of this case is not yet complete, but a transverse cut of the muscular tissue shows that the intramuscular abscesses owe their existence to these organisms. It is, therefore, hardly erroneous to suppose that these fungi, following the interstices of the conjunctive tissue and the lymphatics, found their way into the stump and the body generally.

Finally, Dr. Orth presented himself as an involuntary test-object. In opening the spinal column of a corpse, with extensive sacral decubitus, he inflicted a small wound on the first joint of his left thumb. In spite of immediate cauterization with acid, the wound became quite painful—its edges red, the whole joint swelled, and all phenomena of an infection made their appearance. Next morning, a small quantity of sero-purulent fluid had accumulated under the glued-up edges of the wound. A microscopic examination of it exhibited small chains of bodies very similar to the microsporon, and subsequent cultivation of these organisms from this preparation confirmed their entire identity with the forms above described.—*Berliner Klinische Wochenschrift, No.* 33, 1872.

---

### *ON THE OCCURRENCE OF BACTERIA IN THE DIPHTHERITIC FORMS OF PUERPERAL FEVER.*

Four observations of cases of diphtheritic puerperal fever gave Waldeyer (*Archiv for Gynækologie* iii, 2) an opportunity to examine the exudates in this disease for bacteria, as heretofore these examinations had been confined to the fluids of the uterus, and the blood itself, where Mayrhofer, Cozc and Feltz discovered these organisms. Waldeyer examined the diphtheritic interstitial deposits in the uterine cavity, the puriform masses in the lymphatic vessels of the uterus and the broad ligaments, the peronitic exudate, and in one case also the pleural and pericardial fluid, and found bacteria everywhere—partly between the pus corpuscles and the necrotic tissue elements, partly within the pus corpuscles, and then always in such abundance that the puriform contents of the lymph currents, with the pus corpuscles, consisted largely of bacteria. These all belonged to the ball-bacteria of Cohn.

Waldeyer is not ready to connect the bacteria intimately with the etiology and pathology of the diphtheritic puerperal diseases, but considers them an aggravating movement in their course and issue; as nephritis bacteridica (Klebs, *Recklinghausen* v), has proven that bacteria act as instigators of inflammations. The dark, granulated masses of Virchow in parametritis diphtheritica, as well as the yellow substances in what have so far been described as lymph thrombi engaged in puriform decomposition, will very probably, according to Waldeyer's opinion, be yet explained as the effects of large masses of bacteria.—*Berliner Klinische Wochenschrift, No.* 34, 1872.

---

*ON THE TREATMENT OF PUERPERAL PARAMETRITIS WITH HYDRARGYRUM MURIAT. CORROSIVUM. By Dr. P. GROSSMAN, Assistant Physician to the Gynæcological Clinic of Professor Dr. Spiegelberg, at Breslau, Germany.*

Amongst a large number of puerperal affections, principally parametritis, which occurred in the wards of the above-named clinic, evidently without any local causes, during the winter of 1871–2, and which seemed to be principally confined to those females whose deliveries had taken the most normal course, and who had, in the majority of cases, not sustained any, or at least very slight, lacerations of the soft parts, the author gives the full history and treatment of two very severe, uncomplicated cases, as illustrations. We will follow him through the history of one of them in full, and close with a resume of the facts gained by the observation and treatment of all these cases generally.

Mrs. J. R., primipara, æt. twenty-four, was normally delivered one and a half years previously; admitted at the fortieth week of utero-gestation; labor lasted ten to eleven hours; sustained a very slight epithelial abrasion on the posterior margin of the introitus vaginæ, but otherwise not the slightest laceration. She had been examined only twice since her admission, with the greatest caution. The child, fully matured and well developed, but not nursed by the mother, her mammæ were subjected to slight compression. About twenty to thirty hours post-partum, while quietly resting in bed, she was taken with intense febrile heat.

Six o'clock P.M.—Pulse 120; temperature 39.7 C. (103.4 F.) in the axilla; great thirst; no appetite, and permanent pain in the iliac regions, while the hypogastrium was comparatively free; the same condition prevailed in the objective evidences of pain; the enlarged and painful broad ligaments could be distinctly felt through the flabby abdominal integuments; lochia normal; vagina free of disease; cervix only very slightly torn; phlegmone of both lateral ligaments. Ten leeches were applied to the iliac regions; cataplasms afterward, and internally pills containing each 0.01 gramme (1-10 grain) corrosive sublimate every hour; took only four pills, as she slept some during the night.

Next day (January 29th) pulse, temperature and general condition the same. The local abstractions of blood had not diminished the fever, as even the usual morning remission was not perceptible. Cataplasmata and pills continued every hour until ten had been taken; afterward one only every two hours.

Six o'clock P.M.—Pulse 124; temperature 40.3 C. (104.3 F.) Several dejections during the night.

Next morning (the 30th of January), the patient felt much better; the abdominal pains not felt as long as a quiet position was maintained; thrombus in the middle third of the vena saphena magna dextra, which caused a good deal of pain; pulse 84; temperature 38.0 C. (100.0 F.) Treatment as before.

January 31.—Abdomen less painful—feels well; mammæ slightly swelled; pulse 88; temperature 38.2 C. (100.9 F.); the sublimate treatment discontinued at noon; patient has taken 0.25 gramme (3.7 grains) in all; has had no alvine discharges for thirty-six or forty hours. Pulse in the evening 98; temperature 38.7 C. (101.6 F.); several large evacuations during the night.

February 1.—Abdomen not painful on pressure; exudates much smaller; appetite reappearing; pulse 84; temperature

37.7 C. (99.8 F.), which, by six o'clock P.M., sank to 78 and 37.3 C. (99.1 F.) respectively; no diarrhœa.

February 2.—Still better.

February 3.—Exudates hardly perceptible; nammæ natural; the thrombosed vein slightly tender to the touch; appetite good; all other functions natural.

Twelve days post-partum, and eleven days after the commencement of the disease, the patient was completely cured, and has remained well since.

The history of the second case—one of metritis and parametritis duplex—is very similar to that of the first, with the difference that the temperature and pulse, at the commencement, reached, respectively, 40.0 C. (104.0 F.) and 136 per minute; that the exudates were very large; and that a deep perineal rupture, which had been united by three perineal and three vaginal sutures, healed per primam intentionem. The treatment was strictly the same; the patient took, from the 6th to the 9th of March, 0.3 gramme (4.5 grain) sublimate, and the success of the treament was just as striking. No salivation, or any other unpleasant gastric or intestinal symptom, occurred in either case.

Amongst the general observations made during this epidemic of puerperal parametritis, the following deserve attention:

Whenever the commencement of the disease fell within the first five days after delivery, the febrile symptoms were very intense, but of short duration, as generally, on the fourth, fifth or sixth day, pulse and temperature returned to their normal standard, and within ten to fifteen days, the exudations had disappeared, and all the patients falling sick at that early period were discharged well within that time, with the exception of three, who died with peritonitis. In those cases which commenced later than the seventh day after delivery, the disease lasted from three to four weeks, and even after that time resistant strings and hard portions of tissues, from the vagina upward and around the womb, were still perceptible. Whether the cause of this more rapid resorption of the exudates in cases occurring early, and the slower one in later cases, is owing to their more sero-albuminous character in the former, and their purulent-fibrinous character in the latter, the author is unable to decide. The fact that the exudation in the early cases could be discovered externally, in the late ones only by examination per vagina, was certainly owing to the different positions of the womb and of the broad ligaments in the earlier and later days after pregnancy.

The corrosive sublimate, after from ten to twelve pills had been given within from twenty-four to forty-eight hours, generally caused a marked diminution of the fever, and it was not often necessary to continue its use longer, or to resume it again on account of later paroxysms of fever. A few small doses of tinct. opii, followed by oleum ricini, were generally sufficient to allay any inclination to diarrhœa following its use in a few instances. Cataplasmata were continued as long as exudations remained.

In order to determine whether the febrile phenomena and the exudations would disappear quicker under the administration of the corrosive sublimate than without it, lighter cases were treated without it, and others again without leeches, only by sublimate and cataplasmata; and a careful comparison of the respective results of these different modes of treatment resulted in a decidedly affirmative answer to this question. The longer duration of the fever and the exudative swellings were also observed in two severe cases, where profuse diarrhœa and salivation, after a very few doses of this remedy, forbid its further use. In a large majority of cases, however, these symptoms did not occur to any extent whatever, even after larger doses of sublimate, and the appetite generally returned soon after the cessation of fever.

The author has ceased to use calomel in these cases, as it had to be generally suspended early on account of diarrhœa following its use, without shortening perceptibly the duration of this affection.

After these results, the author considers himself justified to recommend this remedy to the profession generally for further trial in this disease.—*Berliner Klin. Wochenschrift, No.* 34, '72.

*REPORT ON TWO EPIDEMICS OF DIPHTHERIA.*

This report coontains interesting observations on the etiology and treatment of diphtheria, made by a physician in one of the rural districts of Northern Germany—Dr. Koeniger, at Westerde (Oldenburg.)

Two cases of the disease occurred in October, 1868, in two children and a girl of eighteen years. In March, 1869, the disease reappeared in numerous and often severe cases, and twelve patients died during this month and April—amongst them, a female of twenty-four years and a boy of twelve; the rest were children from three to eight years old. The disease spread from child to child at school, and remained confined to the children of certain schools. It generally could be observed, that children who had their seats at school near each other were affected in groups, while more distant scholars remained well. Fifty scholars were treated.

He speaks of the remarkable difference in the degree of severity of the disease in the individual cases occurring, sometimes simultaneously, in one family. Some children were so slightly affected that the diphtheritic exudations had already disappeared on the third day, while others perished as rapidly with decomposition of the blood and uræmia. In some cases, paralytic phenomena occurred in children whose parents were not aware, up to that time, that there was anything wrong with their children's throats.

He considers diphtheria primarily a local disease, which sometimes sooner, sometimes later, causes an infection of the blood, and which is principally scattered abroad by those light cases that are sometimes hardly discovered.

Albuminuria generally, and several times hæmaturia—and also, in several suddenly fatal cases, entire suspension of the secretion of the kidneys—were observed. One boy, after a severe hæmaturia and dropsy had subsided, died (apparently doing well) very suddenly. Two children died under uræmia and spasmodic phenomena. One woman of twenty-five years, with diphtheritic stomatitis without pharyngeal affection, but with albuminuria, finally died under symptoms which might be designated acute chlorosis, without hæmaturia. Three children died with laryngitis diphtheritica.

His treatment, during the first epidemic, consisted principally in the use of chlorate of potash internally and as a gargle; also liquor ferri locally, and iron, wine and quinine internally. This treatment did not seem to have any favorable effect. All the cases commencing with extensive local affections, or being complicated with severe kidney disease, terminated fatally.

From May, 1869, until October, 1871, diphtheria did not occur. In October, 1871, one two-year-old boy died with diphtheritic laryngitis, and his sister, nine years old, who slept in the same bed with him, was taken with diptheria of the external genitals and the urethra. The disease was brought to a favorable termination under the local use of lime water and carbolic acid, and the occurrence of an accommodation paralysis of the eyes, a few weeks afterward, confirmed the correctness of the diagnosis.

From December, 1871, to March, 1872, the disease raged epidemically. About one hundred children were treated, of which twenty-eight, from one to eleven years old, died. In neighboring communities, numerous cases occurred also; and fourteen, ten, fourteen, and thirty-five children died respectively in different small villages. The epidemics emanated everywhere from the schools, and whenever they were closed, the further extension of it was perceptibly checked. The younger children and scholars of the lower classes became sick first, and generally about eight days afterward, the older children, and often all the children belonging to a family. In one of the villages belonging to the district of our reporter, scarlatina prevailed in 1864, and, remarkably enough, not a single child in the place which had had that disease then, had diphtheria in 1881. Should this be accidental, or does a previous attack of scarlatina create immunity from diphtheria?* Scarlatina and diphtheria are apparently kindred, but not identical diseases; they have the kidney and paralytic affections in com-

* I have seen scarlatina and diphtheria, in one limited epidemic in the northern part of Georgia, occur simultaneously in families. The children who had the one remained free from the other disease.—Translator.

mon together, while the high grade of fever is peculiar to scarlatina. The contagion of scarlatina is evidently much more volatile than that of diphtheria.

The more fixed and less volatile nature of the diphtheritic contagion causes the great danger, that diphtheria, in localities where it has once made its appearance, becomes endemic; so that the disease would be hard to get rid of in larger cities.

A large majority of the patients consisted of children from two to ten years old; grown persons were rarely taken, and evidently require a very intense communication with patients of that kind to become affected. Only three of the mothers of diphtheritic children took the disease. Suckling babes remained free in both epidemics—perhaps on account of the frequent swallowing, which may prevent the attachment of the germs of the disease in the throat. The author considers the remarkably good subjective feeling of the patients at the commencement of the disease, even where a large local affection prevails, one of its peculiarities, in contradistinction to the severe subjective phenomena appearing at the commencement of the acute exanthematous diseases.

Albuminuria and kidney affections predominated as complications, also, in this epidemic as they did in the first; in several cases, uræmia, with complete suspension of the secretion of the kidneys; general dropsy of the cavities of the body in one; petechial diathesis of the blood, with sopor, in another; obstruction of the glottis in six cases was the cause of death. Several children died quite unexpectedly when convalescent, and paralytic affections of different character followed a number of cases, and three died from central paralysis.

Dr. Koeniger also expresses, from his experience, a conviction that cases of aphthose angina are frequently mistaken for light cases of diphtheria. He has seen a number of cases of that disease where the small ulcerations on the tonsils were covered with a grayish-white coating; but the invariable occurrence of the same small white ulcerations on the tongue and in the cavity of the mouth generally secures the diagnosis. This disease is also contagious, but yields readily to solution of chlorate of potash.

The success of his treatment in the last epidemic was much more satisfactory than in the first. He ascribes this favorable change to the local application of aqua calcis and of carbolic acid, and pronounces the use of both remedies a decided progress in the treatment of the disease. He uses aqua calcis with aqua distil. (one to four) for gargles, injections into the nostrils, and inhalations, with a few drops of a solution of carbolic acid (acid carbol. pur one part to aqua coloniensis ten parts) to the gargles and injections. The children must gargle very often. The diphtheritic patches in the throat, pharynx, velum palati within reach, are painted over every two or three hours with the above-mentioned solution of carbolic acid; where the nose is diseased, diluted aqua calcis, with a few drops of the solution of carbolic acid are injected through both nostrils, in spite of the struggles of the little patients. These injections have the advantage that they reach the back walls of the throat and soft palate more effectually than any other method of application, and they are considered very essential to success. Solution of chlorate of potash, in mucilaginous vehicles, is administered internally.

In diphtheritic laryngitis, inhalations of diluted aqua calcis (one to four as above) are made persistently every hour—twenty-four times during the day and night—while chlorate of potash is continued internally. Emetics are only sparingly resorted to in cases of extreme danger of suffocation; but the atmosphere of the room, or of a small closet formed round the patient's bed by walls of thick drapery, is kept permanently impregnated by vapors of hot water (steam) as long as the laryngeal troubles continue. When asphyxial symptoms set in, the inhalations and the vapor atmosphere are not tolerated any longer, and tracheotomy remains as the last resort. The lives of two boys were saved by continuing this treatment persistently for over one weak. Whenever, in cases of diphtheria, the voice becomes hoarse, this treatment should be energetically and persistently used to check the angina, and to prevent the further development of diphtheritic membranes in the larynx.

The early use of the lime water and carbolic acid proved decidedly advantageous in severe cases of local diphtheria, in preventing the general affection; after uræmia or septicæmia once take place, no treatment will be of any service.

Our reporter's resume, at the conclusion of his article, designates diphtheria as an epidemic disease, which is principally propagated at school. The cause of the disease is a contagion of not very diffusible character, which primarily affects the mucous membrane of the choanæ or the throat. Local treatment of the diseased portions of these parts with lime water and carbolic acid, as stated above, is frequently successful in checking the progress of the disease, and its influence upon the blood. Early closure of schools in which the disease shows itself adds materially to the restriction of its epidemics. A solution of carbolic acid in cologne water, as above stated, added to the drinking water, and used frequently as a gargle by the unaffected children, is recommended as a prophylactic.—*Berliner Klinische Wochenschrift, No.* 34, 1872.

